# Development and validation of a machine learning-based prediction model for emergence agitation in geriatric patients undergoing orthopedic surgery: a prospective cohort study

**DOI:** 10.3389/fmed.2026.1903005

**Published:** 2026-10-14

**Authors:** Ping Wang, Jinxuan Zhao, Jinhui Huang, Yingying Tang, Jiayi Liu, Xin Wei, Mengshu Zhang

**Affiliations:** 1 School of Nursing, Jinzhou Medical University, Jinzhou, Liaoning, China; 2 The First Affiliated Hospital of Jinzhou Medical University, Jinzhou, Liaoning, China

**Keywords:** emergence agitation, geriatric patients, machine learning, orthopedic surgery, prediction model, risk factors

## Abstract

**Objective:**

This study aims to identify factors associated with emergence agitation in geriatric patients undergoing orthopedic surgery and to develop a machine learning model for predicting the risk of emergence agitation.

**Methods:**

This prospective cohort study included 612 geriatric patients who underwent elective orthopedic surgery at the First Affiliated Hospital of Jinzhou Medical University in Liaoning Province between April 2025 and April 2026. The patients were randomly divided into a training set (*n* = 430) and a test set (*n* = 182) at a 7:3 ratio. Least absolute shrinkage and selection operator (LASSO) regression and multivariable logistic regression analysis were used to identify predictors. Six machine learning algorithms were employed to develop prediction models. Model performance was evaluated using the area under the receiver operating characteristic curve (AUC), calibration curve, and decision curve analysis (DCA). The optimal model was interpreted using Shapley Additive Explanations (SHAP).

**Results:**

The incidence of emergence agitation among geriatric patients who underwent orthopedic surgery was 27.61%. Preoperative anxiety, preoperative cognitive function, dexmedetomidine use, intraoperative hypothermia, intraoperative blood loss, and postoperative NRS pain score were identified as key predictors. The random forest (RF) model demonstrated the best performance, achieving an AUC of 0.905, accuracy of 0.819, sensitivity of 0.820, specificity of 0.818, F1 score of 0.713, and Brier score of 0.111 on the test set. DCA showed that the model had the highest clinical net benefit. SHAP analysis revealed that intraoperative blood loss was the most significant predictor, followed by postoperative NRS pain score and preoperative cognitive function.

**Conclusion:**

We developed and internally validated six machine learning models to predict the risk of emergence agitation in geriatric patients undergoing orthopedic surgery. The RF model achieved the best performance. Following successful external validation in multicenter cohorts, the model may assist clinicians in identifying patients at increased risk of emergence agitation during early PACU recovery and in prioritizing intensified monitoring and timely supportive management.

## 1 Introduction

In the context of global population aging, the progressive decline in physiological functions among older adults, along with a high incidence of degenerative spinal and joint diseases and falls, has led to a continuous increase in the number of geriatric patients undergoing orthopedic surgery (1). Notably, the dense vascular and nerve distribution in orthopedic surgical sites, the relatively large surgical wounds, prolonged intraoperative positioning, and severe postoperative pain contribute to increased sensitivity to various stimuli, making patients highly susceptible to hyperexcitability and even agitation during the emergence period (2). Furthermore, geriatric patients often have multiple comorbidities and age-related decline in organ function, resulting in reduced compensatory reserve and stress response capabilities (3). This reduces their tolerance to anesthetic agents, further increasing the risk of emergence agitation (4).

Emergence agitation, a common complication following general anesthesia, is an acute, self-limiting state of psychomotor excitation that occurs during the emergence period, primarily characterized by confusion, restlessness, disorientation, and purposeless limb movements (5, 6). Although typically lasting from a few minutes to several tens of minutes, emergence agitation can have serious clinical consequences. During episodes of agitation, patients may experience severe adverse events such as surgical wound dehiscence, unplanned extubation, bed falls, and injury to healthcare personnel (7, 8). Furthermore, the intense sympathetic activation induced by agitation can cause marked tachycardia and significant blood pressure fluctuations, thereby increasing the risk of cardiovascular and cerebrovascular events (9). This condition not only prolongs the length of stay in PACU and the total hospital stay, increasing healthcare resource consumption, but also adversely affects surgical outcomes and patient prognosis, ultimately impeding the achievement of enhanced recovery after surgery (ERAS) goals (10, 11). Therefore, early screening and accurate risk assessment of emergence agitation in geriatric patients undergoing orthopedic surgery are crucial for ensuring perioperative safety and improving the efficiency of the PACU.

Although risk factors for emergence agitation have been investigated in previous studies, the majority have focused on pediatric patients and those undergoing thoracic surgery (12, 13). Large-sample, high-quality studies specifically targeting geriatric orthopedic patients, who are at high risk, remain relatively limited. Existing evidence is largely confined to univariable analyses or small-sample retrospective designs (2, 14). Moreover, traditional risk prediction models mainly rely on linear approaches such as logistic regression, which are often incapable of capturing complex nonlinear interactions among variables, thus limiting their predictive accuracy (15).

In recent years, machine learning, a key branch of artificial intelligence, has been increasingly applied in the field of medical risk assessment and prediction. Compared with traditional statistical methods, it offers distinct advantages in handling high-dimensional, complex clinical data and capturing nonlinear relationships (16). However, the application of machine learning to predicting the risk of emergence agitation in geriatric orthopedic surgery patients remains largely unexplored.

In this context, this study employed multiple machine learning algorithms to develop and internally validate a risk prediction model for emergence agitation in geriatric patients undergoing orthopedic surgery, with the intended clinical application as an early PACU risk-stratification tool. By comparing the performance of each model, the optimal model was identified, and its predictive value was preliminarily evaluated, laying the foundation for future multicenter external validation and clinical translation.

## 2 Materials and methods

### 2.1 Study design and participants

This prospective cohort study enrolled 612 geriatric patients who underwent elective orthopedic surgery at the First Affiliated Hospital of Jinzhou Medical University in Liaoning Province between April 2025 and April 2026, using a convenience sampling method. Inclusion criteria: (1) elective orthopedic surgery under general anesthesia; (2) age ≥ 60 years; (3) provision of informed consent and voluntary participation in the study; (4) American Society of Anesthesiologists (ASA) physical status I-III; (5) transfer to the post-anesthesia care unit (PACU) for routine postoperative monitoring. Exclusion criteria: (1) severe visual or hearing impairment that prevented understanding or cooperation with the assessments; (2) a history of alcohol dependence or drug abuse; (3) comorbid dementia or psychiatric disorders; (4) a history of long-term use of sedatives or analgesics; (5) severe dysfunction of the heart, liver, kidneys, or lungs.

This study was reported in accordance with the TRIPOD+AI statement (17) for clinical prediction model studies. The completed TRIPOD+AI checklist is provided in Supplementary Table S1. In addition, a structured assessment of risk of bias and applicability was conducted using the Prediction model Risk of Bias ASsessment Tool (PROBAST) (18), with the detailed domain-level assessment presented in Supplementary Table S2. The flow chart of this study is illustrated in Figure 1.

**Figure 1:**
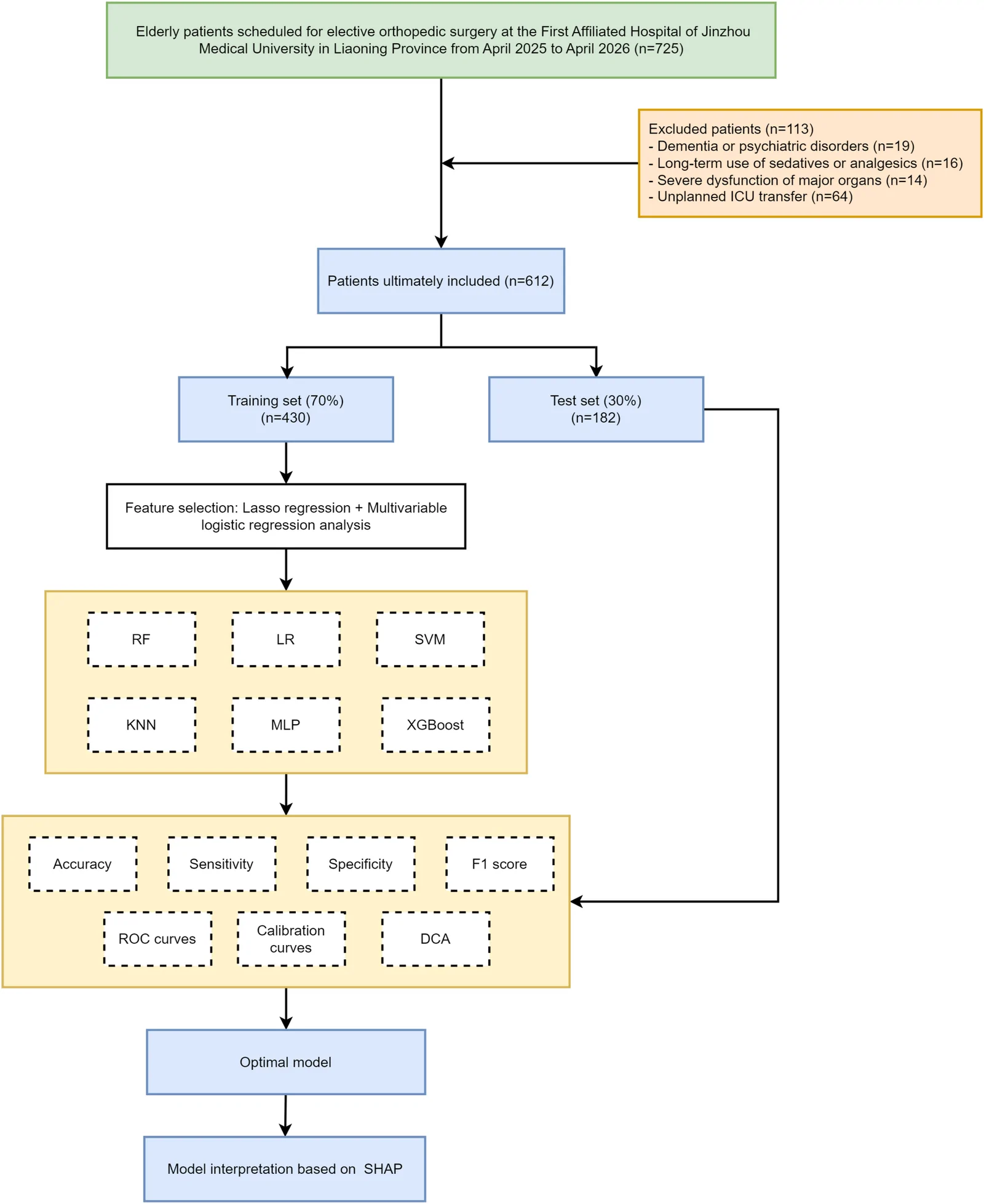
Flow chart of the study.

This study was approved by the Ethics Review Committee of Jinzhou Medical University (JZMULL2025414). All procedures were conducted in accordance with the 1964 Declaration of Helsinki. Prior to the start of the study, written informed consent was obtained from all participants, who voluntarily signed the consent form.

### 2.2 Sample size

Sample size calculation followed the principle of events per variable (EPV) ≥ 10 (19). It was anticipated that no more than 10 predictor variables would be included in the final model. Based on previous studies reporting an incidence of emergence agitation in geriatric patients of 28.7% (20), and accounting for a 10% sample attrition rate, the minimum sample size required for the training set was calculated to be 388. Since the training set accounted for 70% of the total sample, the total sample size was estimated to be at least 555 cases. Ultimately, a total of 612 patients were enrolled in this study.

### 2.3 Study tools

#### 2.3.1 General information questionnaire (GIQ)

This questionnaire was developed by the researchers based on a review of the relevant literature, and it covers the following three aspects. The patient-related data included gender, age, body mass index (BMI), hypertension, diabetes, coronary heart disease, preoperative hemoglobin, and preoperative albumin; the anesthesia-related data included ASA physical status classification and dexmedetomidine use; and the surgery-related data included operative time, type of surgery, intraoperative hypotension (defined as mean arterial pressure < 65 mmHg or a decrease in systolic blood pressure > 20% from baseline for at least 1 min) (21), intraoperative hypothermia (core temperature < 36.0 °C) (22), intraoperative blood loss, and indwelling urinary catheter.

#### 2.3.2 Riker sedation-agitation scale (SAS)

The SAS was developed by American scholar Riker (23) et al. in 1999 to assess sedation and agitation in intensive care unit (ICU) patients and those emerging from anesthesia. It classifies patients into seven continuous levels based on their behaviors and responses to varying degrees of verbal and physical stimulation. Scores range from 1 to 7, representing patients from “unarousable” to “dangerous agitation”. Higher scores indicate more pronounced agitation, and a score of ≥ 5 is generally considered indicative of emergence agitation.

#### 2.3.3 Generalized anxiety disorder 7-item scale (GAD-7)

The GAD-7 was developed by American psychiatrist Spitzer (24) et al. in 2006 to assess the severity of anxiety. It is a unidimensional scale comprising 7 items rated on a 4-point Likert scale. The total score ranges from 0 to 21, with a higher score indicating greater levels of anxiety. In this study, the Cronbach's *α* for this scale was 0.804.

#### 2.3.4 Mini-Cog

The Mini-Cog was developed by Borson (25) et al. in 2000 to assess cognitive function in older adults. The scale consists of two components: a three-word recall task and a clock-drawing test. The specific assessment procedure was as follows: First, the examiner ensured that the patient was fully attentive, and instructed the patient to listen carefully and remember three unrelated words. Next, the clock-drawing test was administered, which was scored as 2 points for a completely correct drawing and 0 points for any errors. Finally, the patient was asked to recall the three words mentioned earlier, with 1 point awarded for each correctly recalled word. The total score ranges from 0 to 5, with a score of < 3 indicating cognitive impairment and a score of ≥ 3 indicating normal cognitive function. In this study, the Cronbach's *α* for the Mini-Cog was 0.797.

#### 2.3.5 Numerical rating scale (NRS)

The NRS was developed by Jensen (26) et al. in 1986 to assess an individual's subjective pain intensity. It is an 11-point numerical rating scale ranging from 0 to 10. Based on their subjective experience, patients select a number from 0 to 10 to describe their pain intensity. A higher number indicates more severe pain.

### 2.4 Data collection

Before data collection, all researchers underwent standardized training to ensure the quality of the survey. One day before surgery, the researchers conducted face-to-face interviews with the patients. Given that some patients might have difficulty understanding the questions, researchers read each item to facilitate completion of the questionnaire. Completed questionnaires were reviewed immediately by the researchers, and any missing responses or logical inconsistencies were promptly addressed and corrected. Information regarding intraoperative anesthesia and surgery was extracted in real time from the anesthesia information management system and the electronic medical record system. After the patients were transferred to the PACU, postoperative pain was assessed by anesthesia nurses within 5 min of admission. Emergence agitation was first assessed 10 min after PACU admission and was subsequently assessed at 10 min intervals throughout the PACU stay. If the SAS score was ≥ 5 at any assessment time point, the patient was considered to have developed emergence agitation.

### 2.5 Data preprocessing

The proportion of missing data was first assessed for each variable. No variable had a missing rate exceeding 20%; therefore, none were excluded. For continuous variables, those with less than 5% missing data were imputed using the mean (for normally distributed variables) or the median (for skewed variables). Variables with 5% or higher missing data were handled using multiple imputation by chained equations (MICE). All categorical variables had complete data and therefore did not require imputation. The specific missing proportions and imputation methods for each variable are detailed in Supplementary Table S3.

After completing the imputation process, continuous variables were standardized using Z-score normalization to eliminate scale effects, yielding a mean of 0 and a standard deviation of 1. For categorical variables, binary variables were label-encoded as 0 and 1, whereas multi-class variables were one-hot encoded to create dummy variables.

To avoid data leakage, all preprocessing steps were applied exclusively to the training set, and the fitted parameters were then uniformly applied to the test set.

### 2.6 Feature selection

In the training set, LASSO regression was used for the initial screening of all predictors. By introducing an L1 regularization term, LASSO regression shrinks the coefficients of unimportant or less contributory variables to zero, thereby achieving feature selection and reducing model dimensionality. Subsequently, the variables selected by LASSO were entered into multivariable logistic regression to identify independent predictors. This two-step screening strategy reduced the risk of false positives in variable selection through sequential validation, thereby enhancing the robustness of the results. The final unified set of predictors was fixed and then applied to all six machine learning models, which eliminated the confounding effect of variable set differences and ensured fairness and comparability in the comparison of model performance across different algorithms.

### 2.7 Model construction and evaluation

This study employed six machine learning algorithms to develop prediction models, including random forest (RF), logistic regression (LR), support vector machine (SVM), k-nearest neighbors (KNN), multilayer perceptron (MLP), and extreme gradient boosting (XGBoost). The dataset was randomly divided into a training set (*n* = 430) and a test set (*n* = 182) at a 7:3 ratio, using stratified sampling to maintain comparable outcome proportions between the two sets. To ensure the reproducibility of the results, a random seed of 123 was set before the split. The training set was used for model training and hyperparameter optimization, whereas the independent internal test set was used for preliminary evaluation of model performance.

During model construction, 5-fold cross-validation combined with grid search was applied to determine the optimal hyperparameters, with the cross-validated average AUC serving as the primary optimization metric. Given the clinical importance of identifying agitation events, a minimum sensitivity constraint was imposed during hyperparameter screening: only parameter combinations with a cross-validated average sensitivity ≥ 0.6 were retained. This constraint prevented the model from becoming overly biased toward the majority class and neglecting the identification of agitation events. Subject to this sensitivity constraint, the hyperparameter combination with the highest cross-validated average AUC was selected as the final model parameters. The hyperparameter search space and the final selected parameters for each algorithm are detailed in Supplementary Table S4.

In this study, the ratio of positive to negative samples was approximately 1:2.6, which is lower than the threshold commonly used for handling imbalanced data in the field of machine learning (1:4). To preserve the true distribution of the original data, oversampling, undersampling, and class weighting strategies were not applied. Minority class identification was improved solely through threshold optimization: the optimal classification threshold was determined by maximizing the Youden index on the training set, and this threshold was then fixed and applied to the test set, thereby mitigating the impact of the default 0.5 threshold on classification performance for the imbalanced outcome.

On the test set, the predictive performance of the models was evaluated by calculating accuracy, sensitivity, specificity, F1 score, and AUC. Calibration was comprehensively evaluated using calibration curves, Brier score, together with quantitative metrics including calibration intercept and calibration slope. Clinical utility was assessed by DCA.

### 2.8 Model interpretability analysis

Machine learning models are not inherently “black-box” or opaque; their interpretability depends on the underlying algorithmic architecture, ranging from intrinsically transparent models (e.g., decision trees and logistic regression) to fully opaque complex models. This spectrum of interpretability aligns with the transparent–translucent–opaque nomenclature for clinical AI proposed by Melvin (27). The optimal model developed in this study is a high-complexity ensemble model that falls into the opaque category and lacks intrinsic interpretability. Therefore, this study introduced the SHapley Additive exPlanations (SHAP) method to conduct an interpretability analysis of the optimal model. This method is based on Shapley value theory from cooperative game theory. By calculating SHAP values to quantify the contribution of each variable, it helps to understand how each variable influences the model output (28).

### 2.9 Statistical analysis

Statistical analysis was performed using IBM SPSS Statistics 25.0 and R 4.5.1. Normally distributed continuous variables were presented as mean ± standard deviation, and the *t*-test was used for comparisons between groups. Non-normally distributed continuous variables were presented as median with interquartile range, and the Wilcoxon rank-sum test was applied for comparisons between groups. Categorical variables were expressed as frequencies (percentages), and comparisons between groups were performed using the chi-square test or Fisher's exact test. A two-sided *p* value < 0.05 was considered statistically significant.

## 3 Results

### 3.1 Baseline characteristics

A total of 612 patients were included in this study, with 169 patients developing emergence agitation, yielding an incidence of 27.61%. The incidence of emergence agitation was 27.67% in the training set and 27.47% in the test set. There were no statistically significant differences in patient characteristics between the training and test sets (*p* > 0.05), indicating that the two groups were balanced and comparable. The detailed characteristics of the dataset are shown in Table 1.

**Table 1:** Comparison of data variability between training and test datasets.

Variables	Total (*n* = 612)	Train (*n* = 430)	Test (*n* = 182)	*p*
Gender				0.409
Male	217 (35.46)	148 (34.42)	69 (37.91)	
Female	395 (64.54)	282 (65.58)	113 (62.09)	
Age (years)	70.00[65.00,75.00]	70.00[65.00,75.00]	70.00[65.00,75.00]	0.894
BMI (kg/m^2^)	23.88[21.46,26.37]	23.88[21.26,26.40]	23.88[21.59,26.25]	0.678
Hypertension				0.652
No	328 (53.59)	233 (54.19)	95 (52.20)	
Yes	284 (46.41)	197 (45.81)	87 (47.80)	
Diabetes				0.549
No	467 (76.31)	331 (76.98)	136 (74.73)	
Yes	145 (23.69)	99 (23.02)	46 (25.27)	
Coronary Heart Disease				0.084
No	551 (90.03)	393 (91.40)	158 (86.81)	
Yes	61 (9.97)	37 (8.60)	24 (13.19)	
Preoperative anxiety	8.00[7.00,10.00]	8.00[7.00,10.00]	8.00[7.00,10.00]	0.484
Cognitive function				0.497
Normal	415 (67.81)	288 (66.98)	127 (69.78)	
Abnormal	197 (32.19)	142 (33.02)	55 (30.22)	
Preoperative hemoglobin (g/L)	131.00[119.25,142.00]	131.00[117.00,142.00]	131[121.75,143.25]	0.313
Preoperative albumin (g/L)	40.60[37.73,43.10]	40.80[37.90,43.13]	40.45[37.55,43.15]	0.551
ASA				0.919
Ⅰ	0 (0.00)	0 (0.00)	0 (0.00)	
Ⅱ	459 (75.00)	322 (74.88)	137 (75.27)	
Ⅲ	153 (25.00)	108 (25.12)	45 (24.73)	
Dexmedetomidine use				0.777
No	271 (44.28)	192 (44.65)	79 (43.41)	
Yes	341 (55.72)	238 (55.35)	103 (56.59)	
Operative time (min)	96.00[73.25,120.75]	95.00[70.75,116.00]	100[74.75,132.00]	0.093
Type of surgery				0.535
Joint surgery	264 (43.14)	191 (44.42)	73 (40.11)	
Spinal surgery	210 (34.31)	142 (33.02)	68 (37.36)	
Trauma surgery	138 (22.55)	97 (22.56)	41 (22.53)	
Intraoperative hypotension				0.279
No	516 (84.31)	367 (85.35)	149 (81.87)	
Yes	96 (15.69)	63 (14.65)	33 (18.13)	
Intraoperative hypothermia				0.737
No	520 (84.97)	364 (84.65)	156 (85.71)	
Yes	92 (15.03)	66 (15.35)	26 (14.29)	
Intraoperative blood loss (mL)	60.00[20.00,150.00]	60.00[20.00,150.00]	60[23.75,150.00]	0.234
Indwelling urinary catheter				0.177
No	271 (44.28)	198 (46.05)	73 (40.11)	
Yes	341 (55.72)	232 (53.95)	109(59.89)	
NRS	4.00[3.00,5.00]	4.00[3.00,5.00]	4.00[3.00,5.00]	0.654

### 3.2 Feature selection

In the training set, LASSO regression was used for feature dimension reduction and selection, and the optimal regularization parameter *λ* was determined through 10-fold cross-validation. Given that this study aimed to develop a highly discriminative prediction model, we selected *λ*.min that minimized the cross-validation error to maximize the model's predictive performance. Ultimately, at *λ*.min = 0.013451, 10 variables with non-zero regression coefficients were retained, including gender, BMI, preoperative anxiety, preoperative cognitive function, preoperative hemoglobin, dexmedetomidine use, intraoperative hypothermia, intraoperative blood loss, indwelling urinary catheter, and postoperative NRS pain score (Figure 2). Subsequently, we performed a multivariable logistic regression analysis with these 10 variables as the independent variables and the occurrence of emergence agitation as the dependent variable. The results showed that preoperative anxiety, preoperative cognitive function, dexmedetomidine use, intraoperative hypothermia, intraoperative blood loss, and postoperative NRS pain score were independent predictors of emergence agitation in geriatric patients undergoing orthopedic surgery (*p* < 0.05) (Table 2).

**Figure 2:**
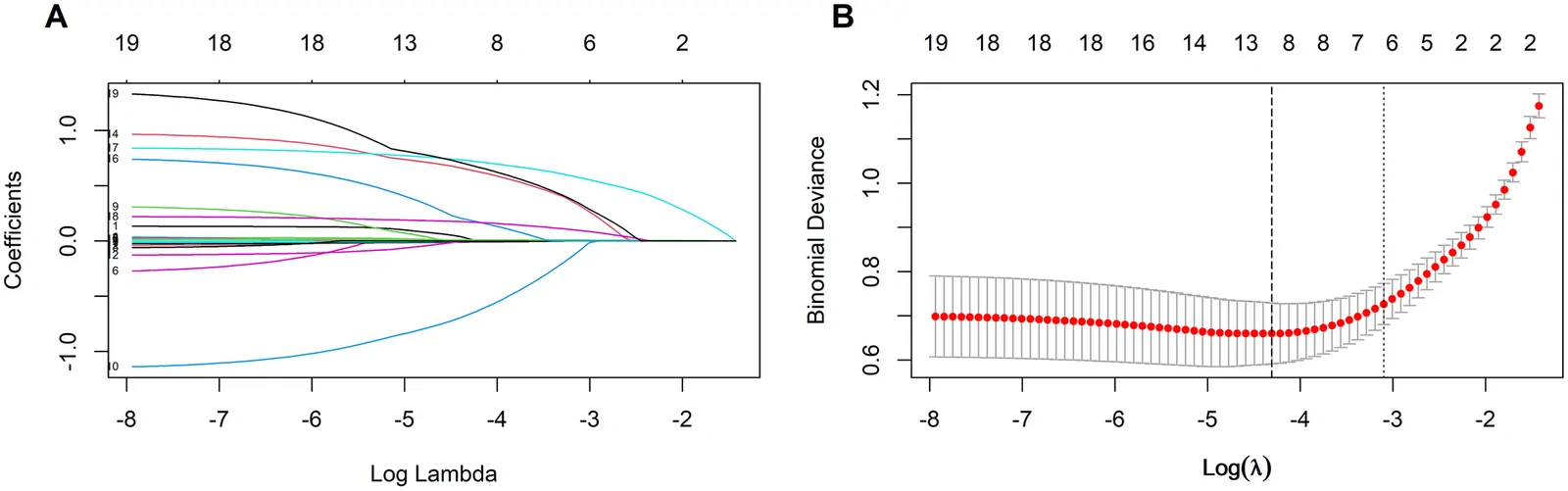
Feature selection using the LASSO regression. **(A)** LASSO coefficient path plot. **(B)** LASSO cross-validation plot.

**Table 2:** Multivariable logistic regression analysis of predictors of emergence agitation in geriatric patients undergoing orthopedic surgery.

Variable	LASSO coefficient	*β*	SE	OR	95% CI	*p*
Gender	0.022	0.276	0.357	1.317	0.656–2.676	0.441
BMI	0.003	0.036	0.044	1.037	0.950–1.132	0.415
Preoperative anxiety	0.174	0.222	0.062	1.249	1.109–1.415	**<0**.**001**
Cognitive function	0.693	0.962	0.333	2.618	1.367–5.073	**0**.**004**
Preoperative hemoglobin	−0.008	−0.015	0.010	0.985	0.966–1.004	0.134
Dexmedetomidine use	−0.665	−1.077	0.332	0.341	0.175–0.645	**0**.**001**
Intraoperative hypothermia	0.647	0.861	0.421	2.366	1.041–5.450	**0**.**041**
Intraoperative blood loss	0.011	0.014	0.002	1.014	1.010–1.019	**<0**.**001**
Indwelling urinary catheter	0.193	0.427	0.398	1.533	0.703–3.373	0.283
NRS	0.729	0.853	0.145	2.347	1.786–3.154	**<0**.**001**

Bold values indicate statistical significance at *p* < 0.05.

### 3.3 Model performance comparison

This study comprehensively analyzed the performance of six machine learning algorithms on the training and test sets across multiple evaluation metrics. As shown in Table 3, the accuracy of the models on the training set ranged from 0.835 to 0.893, sensitivity from 0.899 to 0.941, specificity from 0.810 to 0.891, F1 score from 0.751 to 0.823, and AUC from 0.924 to 0.963. The accuracy of the models on the test set ranged from 0.753 to 0.819, sensitivity from 0.700 to 0.820, specificity from 0.765 to 0.856, F1 score from 0.615 to 0.713, and AUC from 0.857 to 0.905. On the test set, the RF model demonstrated the highest sensitivity and F1 score, suggesting its superior ability to correctly identify patients with emergence agitation.

**Table 3:** Performance comparison of the six models in the training set and test set.

Model	Dataset	Accuracy	Sensitivity	Specificity	F1 score	AUROC(95%CI)
RF	Training	0.863	0.933	0.836	0.790	0.958 (0.940–0.973)
	Test	0.819	0.820	0.818	0.713	0.905 (0.857–0.946)
LR	Training	0.856	0.908	0.836	0.777	0.924 (0.898–0.950)
	Test	0.780	0.740	0.796	0.649	0.877 (0.822–0.924)
SVM	Training	0.861	0.908	0.842	0.783	0.943 (0.920–0.962)
	Test	0.791	0.740	0.811	0.661	0.883 (0.831–0.928)
KNN	Training	0.835	0.899	0.810	0.751	0.938 (0.917–0.957)
	Test	0.753	0.720	0.765	0.615	0.857 (0.797–0.909)
MLP	Training	0.893	0.899	0.891	0.823	0.963 (0.948–0.977)
	Test	0.813	0.700	0.856	0.673	0.877 (0.822–0.925)
XGBoost	Training	0.870	0.941	0.842	0.800	0.956 (0.938–0.972)
	Test	0.813	0.780	0.826	0.696	0.902 (0.853–0.941)

The ROC curves for the training and test sets are shown in Figure 3. All models demonstrated excellent discriminative ability on the training set. On the independent test set, the RF model achieved the highest AUC of 0.905 (95% CI: 0.857–0.946), demonstrating the best discriminative ability. The AUC values of all models on the test set decreased by less than 10% compared to those on the training set, indicating no substantial overfitting.

**Figure 3:**
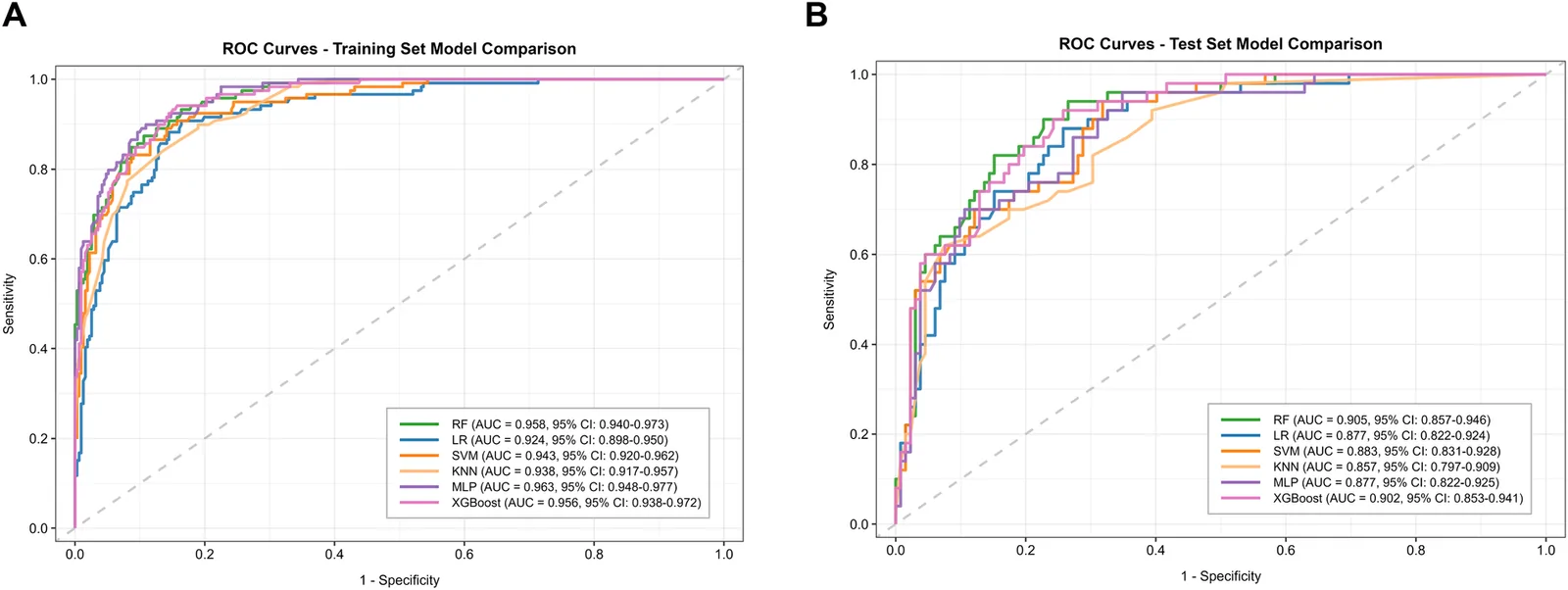
Model ROC curves. **(A)** ROC curves of the six models in the training set. **(B)** ROC curves of the six models in the test set.

Calibration curves were used to assess the consistency between predicted probabilities and actual event rates. The Brier score quantified the calibration error; the lower the score, the better the calibration. Furthermore, the calibration intercept and calibration slope provide a quantitative assessment of systematic bias; ideally, the intercept should approach 0 and the slope should approach 1. On the test set, the calibration curve of the RF model was closest to the ideal diagonal, achieving the lowest Brier score of 0.111. Its calibration intercept was −0.024 and its calibration slope was 1.100. Both metrics are close to the ideal standard as presented in Supplementary Table S5, indicating that the model's predicted probabilities are closest to the true risks. Calibration curves for the training and test sets are shown in Figure 4.

**Figure 4:**
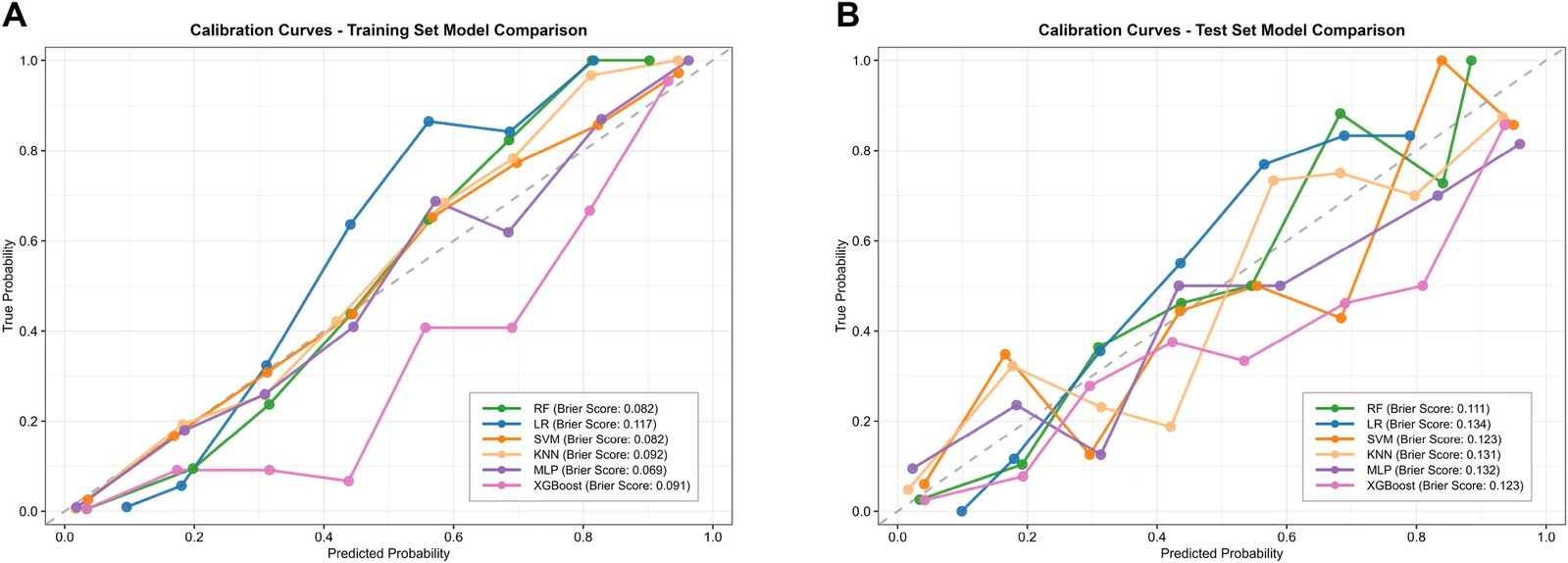
Model calibration curves. **(A)** Calibration curves of the six models in the training set. **(B)** Calibration curves of the six models in the test set.

DCA was used to evaluate the clinical net benefit of different models. On the test set, most models demonstrated greater net benefit than the “treat-none” and “treat-all” strategies when the threshold probability ranged from 20% to 70%, suggesting that the models have good clinical translation potential. Among them, the RF model yielded the highest and most stable net benefit at most threshold probabilities, significantly outperforming the other models. This indicates its greater advantage in identifying high-risk patients who require intervention and ruling out low-risk patients to avoid unnecessary intervention. As shown in Figure 5.

**Figure 5:**
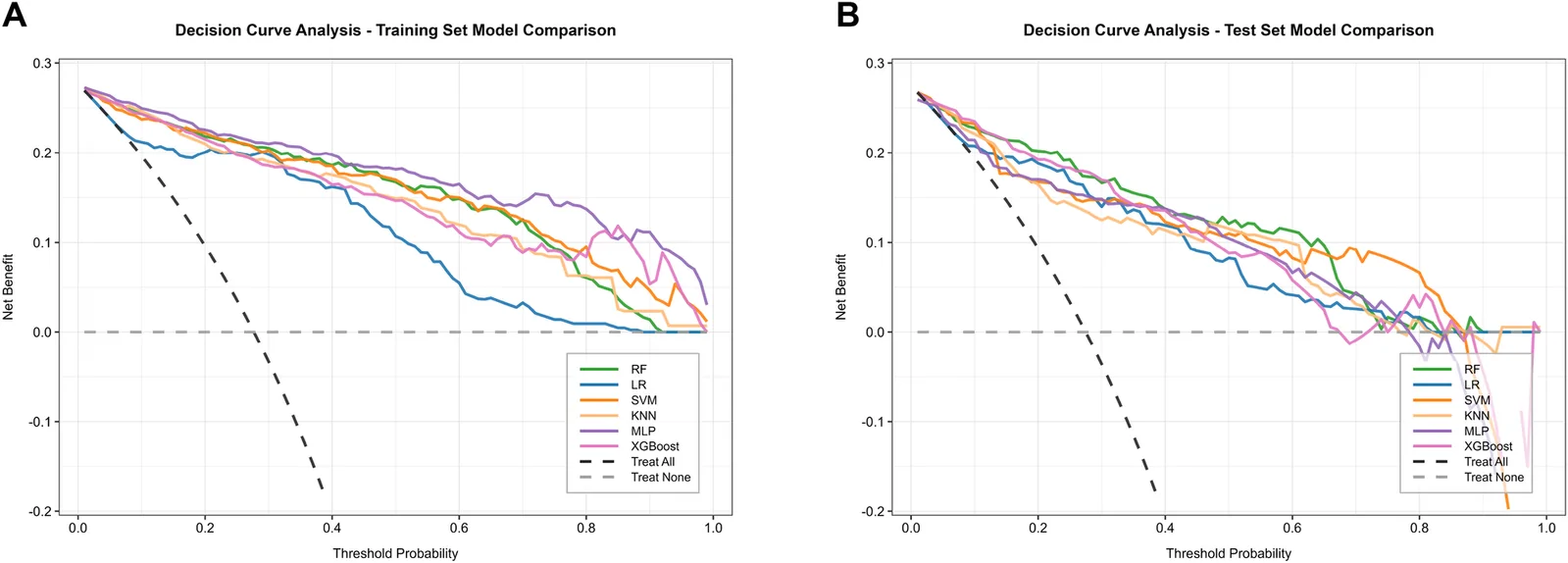
Model DCA curves. **(A)** DCA curves of the six models in the training set. **(B)** DCA curves of the six models in the test set.

In summary, the RF model exhibited outstanding performance in discrimination, calibration, and clinical utility, and was identified as the optimal prediction model.

### 3.4 Model interpretation

To further intuitively explain the influence of the selected variables on the RF model, we employed the SHAP algorithm. The SHAP feature importance plot (Figure 6A) showed that intraoperative blood loss had the highest contribution to RF model predictions, followed by postoperative NRS pain score, preoperative cognitive function, preoperative anxiety, dexmedetomidine use, and intraoperative hypothermia. The SHAP summary plot (Figure 6B) further illustrated the specific influence patterns of the features on the model output. Each point represents a sample, and the horizontal axis displays the SHAP value for each sample; a higher value indicates a greater influence of the feature on the model output (positive values indicate a positive association with higher agitation risk, negative values indicate a negative association with higher agitation risk). The color of the points represents the feature values (yellow indicates higher values, dark purple indicates lower values). The results showed that higher intraoperative blood loss, higher postoperative NRS pain score, impaired preoperative cognitive function, higher preoperative anxiety, and intraoperative hypothermia were positively associated with higher predicted agitation risk to varying degrees, whereas dexmedetomidine use was negatively associated with predicted agitation risk.

**Figure 6:**
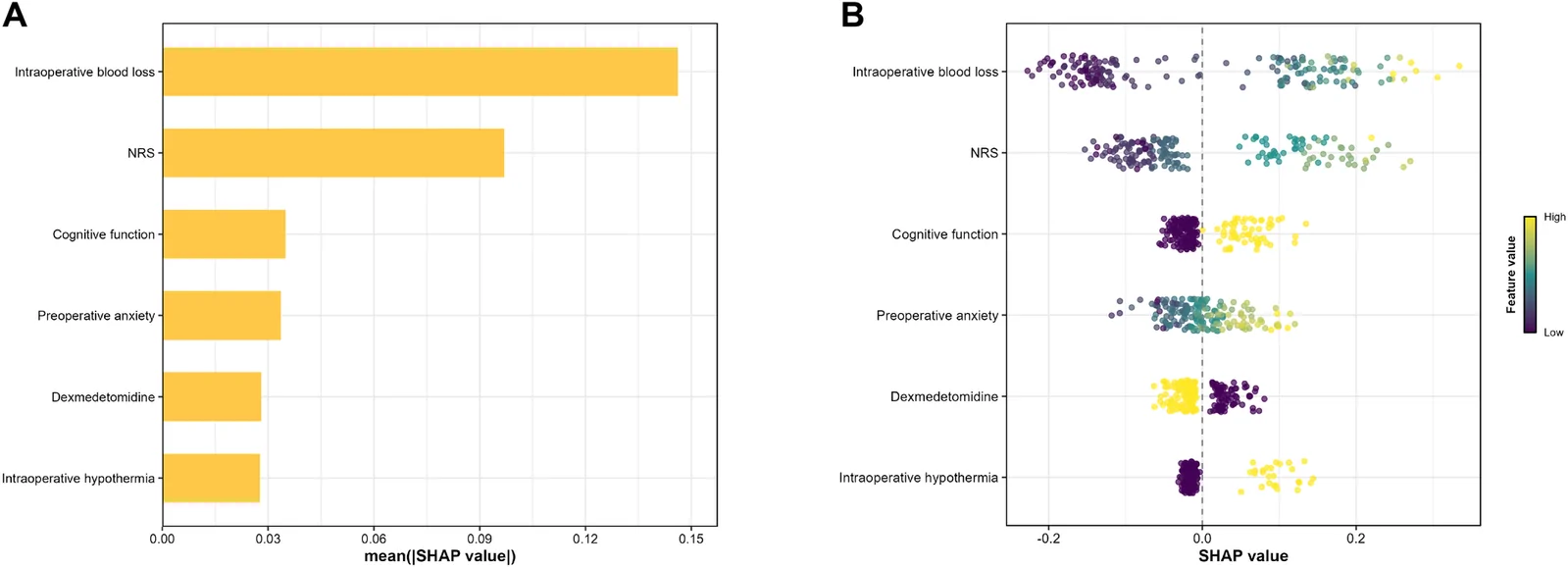
SHAP plot of the RF model. **(A)** SHAP feature importance plot. **(B)** SHAP summary plot.

## 4 Discussion

### 4.1 Model performance analysis

By systematically comparing six mainstream machine learning algorithms, this study developed and internally validated a prediction model to identify the risk of emergence agitation in geriatric patients undergoing orthopedic surgery. On the internal test set, the RF model showed the strongest overall performance among the evaluated models, with an AUC of 0.905, accuracy of 0.819, sensitivity of 0.820, specificity of 0.818, F1 score of 0.713, and Brier score of 0.111. The ability of RF to flexibly capture nonlinear relationships and potential interactions among predictors may partly explain its performance in the present dataset (29).

However, the incremental improvement over simpler approaches was modest and should be interpreted cautiously. The test-set AUC was 0.905 for RF compared with 0.877 for logistic regression, corresponding to an absolute difference of 0.028, while XGBoost achieved a similarly high AUC of 0.902. Thus, although RF performed best numerically, whether this relatively small improvement translates into a clinically meaningful advantage over simpler and more interpretable models remains uncertain.

Previous risk prediction tools for emergence agitation in patients under general anesthesia have mostly relied on logistic regression-based nomograms, with study populations typically encompassing a variety of elective surgery patients. Prediction models specifically targeting geriatric patients undergoing orthopedic surgery are relatively scarce. The reported AUCs of similar nomograms have largely fallen within the range of 0.62–0.82 (30–32). Nevertheless, direct comparisons across studies should be interpreted cautiously because of differences in study populations, predictor sets, outcome definitions, and validation strategies. In the present study, DCA suggested a relatively favorable net benefit for RF across a range of threshold probabilities; however, whether this modest incremental predictive gain translates into improved clinical outcomes or more efficient clinical workflows requires further evaluation. External validation and prospective clinical impact studies are therefore needed to assess the practical value of RF relative to simpler approaches.

### 4.2 Predictors associated with emergence agitation in the context of perioperative brain vulnerability

Contemporary concepts of perioperative brain health emphasize that older patients differ substantially in baseline neurocognitive vulnerability and in their capacity to tolerate acute physiological stress associated with anesthesia and surgery (33, 34). Emergence agitation (EA) occurs during the early recovery from anesthesia and is characterized predominantly by acute behavioral and arousal disturbances, whereas postoperative delirium (POD) is a clinically defined delirium syndrome occurring during the postoperative period (7, 33, 35). Although EA and POD differ in timing, diagnostic constructs, and assessment and should not be regarded as interchangeable, they may share some predisposing and precipitating factors. In older patients, reduced cognitive and brain reserve may limit the capacity to compensate for perioperative disturbances, providing a conceptual basis for understanding how baseline vulnerability may interact with acute perioperative stress and increase susceptibility to transient neurobehavioral disturbances during emergence (34, 36, 37).

Within this vulnerability–stressor framework, preoperative cognitive impairment and anxiety may represent different dimensions of baseline susceptibility. In the present study, impaired preoperative cognitive function was associated with a higher predicted risk of EA. Reduced cognitive and brain reserve may diminish the ability of older patients to maintain cerebral function in the face of transient disturbances in perfusion, metabolism, neurotransmission, and arousal. Studies in older surgical populations have similarly linked lower cognitive or brain reserve with susceptibility to postoperative delirium, supporting the broader concept that pre-existing neurocognitive vulnerability modifies the brain's response to perioperative insults (36, 37). Preoperative anxiety may represent an additional dimension of vulnerability. Heightened anxiety can amplify autonomic and neuroendocrine stress responses and may lower the threshold for behavioral responses to perioperative stimuli (38, 39). Thus, cognitive impairment and anxiety may be clinically relevant not only as individual predictors, but also as markers of reduced resilience to subsequent perioperative stress.

In contrast, intraoperative blood loss, hypothermia, and acute postoperative pain can be considered perioperative physiological stressors acting on this underlying vulnerability. SHAP analysis identified intraoperative blood loss as the feature with the largest contribution to RF predictions. Excessive blood loss may reduce effective circulating volume and cerebral oxygen delivery, while associated metabolic, electrolyte, and acid–base disturbances may further disrupt cerebral homeostasis (40, 41). Older patients may be particularly susceptible to such insults because age-related reductions in physiological reserve and cerebral autoregulatory capacity can limit compensation for acute hemodynamic disturbances. Intraoperative hypothermia represents an additional metabolic stressor that may alter drug metabolism and delay recovery from anesthesia, thereby prolonging disturbances in central nervous system function (32, 42). Acute postoperative pain, which was assessed shortly after PACU admission in the present study, represents a temporally proximal nociceptive stressor. Pain may activate sympathetic–adrenomedullary and other stress-response pathways, increasing arousal and physiological instability during emergence (30, 31, 43). Considered together, these findings suggest that hemodynamic, metabolic, and nociceptive stressors may interact with baseline neurocognitive vulnerability rather than acting through completely independent pathways.

Dexmedetomidine occupies a different position within this framework because its administration represents an intraoperative treatment exposure rather than an intrinsic marker of patient vulnerability or physiological stress. Through *α*₂-adrenergic receptor agonism, dexmedetomidine produces sedative, anxiolytic, analgesic, and sympatholytic effects that may modulate arousal and stress responses during emergence (44, 45). However, dexmedetomidine administration in our cohort was determined by anesthesiologists according to routine clinical judgment rather than by random assignment; therefore, confounding by indication cannot be excluded. Accordingly, its association with lower predicted EA risk should be interpreted as a predictive relationship reflecting intraoperative treatment exposure rather than evidence of an independent causal protective effect. The present model is therefore not intended to guide the decision to administer dexmedetomidine intraoperatively.

Taken together, the pattern of predictors identified in this study is consistent with a vulnerability–stressor framework of acute perioperative brain dysfunction. Pre-existing neurocognitive and psychological vulnerability may reduce resilience to perioperative insults, whereas hemodynamic, metabolic, and nociceptive stressors may further perturb cerebral homeostasis and arousal regulation during emergence. Treatment exposures may additionally modify this trajectory. This integrated framework may help explain why predictors arising at different stages of the perioperative period jointly contribute to EA risk. Importantly, however, this interpretation does not imply that EA and POD are equivalent, nor does it establish reduced cerebral reserve, neuroinflammation, or altered stress responses as causal mechanisms of EA. Rather, it provides a biologically plausible framework for interpreting the observed predictive associations and for generating hypotheses for future mechanistic studies.

### 4.3 Clinical translation and implementation feasibility of the model

Although the RF model demonstrates good risk stratification performance, its clinical value relies on successful integration into routine PACU management workflows. Given that the predictor set includes intraoperative variables (dexmedetomidine use, intraoperative hypothermia, and intraoperative blood loss) and immediate postoperative pain score, we explicitly position this model as an early risk stratification tool for use after the initial PACU assessment, rather than a preoperative screening tool.

The six core predictors incorporated in this model are routinely available shortly after PACU admission: preoperative baseline variables (anxiety and cognitive function) are collected during the preoperative visit; intraoperative variables are available at PACU handover, and the initial postoperative NRS pain score is obtained within 5 min after PACU admission. Thus, all predictors required by the model become available during the initial PACU assessment. The optimal probability threshold was determined by maximizing the Youden index on the training set, yielding a value of 0.303. Accordingly, patients can be divided into a low-risk group (predicted probability < 0.303) and a high-risk group (predicted probability ≥ 0.303). For patients identified as high risk during early PACU assessment, the following measures are recommended: continuous verbal reorientation to reduce disorientation; dynamic pain assessment should target an NRS score < 4, and if this target is not met, the anesthesiologist should be promptly notified and assisted with analgesic administration; intensified bedside monitoring to prevent agitation-related adverse events such as unplanned extubation and falls; and active correction of modifiable risk factors such as residual hypothermia. For low-risk patients, routine monitoring is performed to avoid excessive intervention.

In the future, the model could be interfaced with electronic health record systems to automatically extract perioperative data and generate risk alerts shortly after PACU admission, thereby assisting clinical teams in risk stratification. Nevertheless, clinical implementation faces practical barriers, including clinicians’ skepticism toward predictions from opaque, high-complexity models whose internal decision logic is not directly interpretable. The use of SHAP-based feature attribution analysis to visually present the risk contributions of individual predictors can enhance the transparency of model-driven decisions. Once the stability of the model has been verified via multicenter external validation, pilot operational testing can be conducted in the PACU setting to assess system compatibility. Subsequently, prospective interventional studies should be performed to evaluate whether model-guided PACU nursing improves patient outcomes, facilitating the translation of model into clinical practice.

### 4.4 Limitations

Several limitations should be acknowledged. First, this is a single-center study, and all participants were recruited from one institution, involving only elective geriatric patients undergoing orthopedic surgery. The study population was relatively homogeneous in terms of geographic region, healthcare setting, and case type, and did not cover broader clinical scenarios such as emergency orthopedic procedures or other tiers of healthcare facilities. Consequently, the generalizability of this model to geriatric orthopedic patients in different regions, at different centers, or undergoing different types of surgery remains limited, and extrapolation of our findings should be undertaken with caution. Second, this study only performed internal validation in a single center, and has not yet been externally validated in independent multicenter cohorts. The stability and generalizability of the model in heterogeneous populations have not been fully evaluated, and its clinical applicability warrants further confirmation. Third, the mechanisms underlying emergence agitation are complex. This study included only variables that are readily available in clinical practice, relevant factors such as inflammatory cytokines and intraoperative depth of anesthesia monitoring were not incorporated into the analysis. Fourth, the feature selection process in this study was based on linear assumptions, which may have overlooked predictors that only exhibit nonlinear effects or high-order interactions, thus failing to fully unleash the feature mining potential of nonlinear models such as random forest and XGBoost. This strategy was adopted to balance model robustness and clinical interpretability, at the cost of partially limiting the exploratory capacity of nonlinear models. Finally, given the temporal availability of the included predictors, the model is intended as an early PACU risk-update tool rather than a preoperative risk-prediction model. Because it incorporates intraoperative variables, including dexmedetomidine use, as well as an early postoperative pain assessment, the model cannot be used for preoperative counseling or anesthetic planning and is not applicable to agitation occurring before completion of the initial PACU assessment. Moreover, dexmedetomidine administration was determined by anesthesiologists according to clinical judgment rather than by random assignment; therefore, confounding by indication cannot be excluded, and the observed association between dexmedetomidine use and a lower risk of emergence agitation should not be interpreted as causal. Future studies should include larger, multicenter cohorts covering a broader range of geriatric orthopedic surgical settings and should perform independent external validation to assess model transportability. More comprehensive predictors and refined feature-selection strategies should also be explored. In addition, prospective validation within the predefined early PACU prediction window is warranted to evaluate the model's clinical utility and robustness across institutions with different perioperative management and anesthetic practice patterns.

## 5 Conclusion

In conclusion, this study identified intraoperative blood loss, postoperative NRS pain score, preoperative cognitive function, preoperative anxiety, dexmedetomidine use, and intraoperative hypothermia as predictors of emergence agitation in geriatric patients undergoing orthopedic surgery. We developed six machine learning models to predict the risk of emergence agitation, with the RF model demonstrating the best predictive performance. SHAP analysis rendered the model well interpretable. The model is positioned as an early risk-stratification tool for use after PACU admission, consistent with the temporal availability of all included predictors. However, the above findings are based solely on single-center internal validation. Therefore, future multicenter external validation is necessary to evaluate its stability and generalizability prior to clinical application.

## Data Availability

The data analyzed in this study is subject to the following licenses/restrictions: The datasets generated and/or analyzed during the current study are not publicly available due to patient confidentiality, but are available from the corresponding author upon reasonable request. Requests to access these datasets should be directed to Mengshu Zhang, zhangmengshu@163.com.
