# Supplementary material for: Development and validation of a machine learning-based prediction model for emergence agitation in geriatric patients undergoing orthopedic surgery: a prospective cohort study
Source: Front Med (Lausanne). 2026 Oct 14;13:1903005. doi: 10.3389/fmed.2026.1903005 (PMC13713869; doi:10.3389/fmed.2026.1903005)
Supplement: Supplementary file 2 [file Table2.docx]

**PROBAST assessment of risk of bias and applicability**

**Interpretation note:** PROBAST was designed primarily for assessment of prediction-model studies in systematic reviews. For this author self-assessment, applicability was judged against the study’s stated target population and intended early-PACU use. Y=yes; PY=probably yes; PN=probably no; N=no; NI=no information.

# Step 1. Specify your systematic review question

| Intended use of model | Early PACU risk stratification after the initial PACU assessment, to identify geriatric orthopedic surgery patients at increased risk of emergence agitation and support intensified monitoring and timely supportive management. |
| --- | --- |
| Participants including selection criteria and setting | Adults aged ≥60 years undergoing elective orthopedic surgery under general anesthesia, ASA I–III, transferred to PACU; single-centre prospective cohort at the First Affiliated Hospital of Jinzhou Medical University, Liaoning, China (April 2025–April 2026). |
| Predictors | Candidate set: 19 routinely available perioperative variables. Final common predictor set: preoperative anxiety, preoperative cognitive function, dexmedetomidine use, intraoperative hypothermia, intraoperative blood loss, and postoperative NRS pain score. Preoperative variables were measured before surgery; intraoperative variables were available at PACU handover; NRS was obtained within 5 minutes after PACU admission. |
| Outcome to be predicted | Emergence agitation during PACU recovery, defined as Riker Sedation-Agitation Scale (SAS) score ≥5 at any assessment. Assessment began 10 minutes after PACU admission and was repeated every 10 minutes throughout the PACU stay. |

# Step 2. Classify the type of prediction model evaluation

**Classification: Development only ✓** The study developed prediction models and used internal validation (5-fold cross-validation during tuning plus a random 70/30 split). No external validation in independent participants was performed. Under PROBAST terminology, internal validation remains within “development only”.

| **Publication / study** | **Models of interest** | **Outcome of interest** |
| --- | --- | --- |
| Current manuscript | RF, LR, SVM, KNN, MLP, XGBoost (same cohort, candidate predictors, outcome definition, preprocessing, and internal validation framework; RF selected as optimal) | Emergence agitation during PACU recovery |

# Step 3: Assess risk of bias and applicability

## Domain 1. Participants

**Description:** Prospective single-centre cohort of 612 elective geriatric orthopedic surgery patients meeting prespecified eligibility criteria.

| **Question** | **Judgement** | **Manuscript basis** | **Rationale** |
| --- | --- | --- | --- |
| 1.1 Were appropriate data sources used? | Y | Methods 2.1, p.3 | A prospective cohort is an appropriate data source for prediction-model development. |
| 1.2 Were all inclusions and exclusions of participants appropriate? | PY | Methods 2.1, p.3 | Eligibility criteria are explicit and clinically reasonable. Convenience sampling may limit representativeness but does not by itself establish selection bias within the target setting. |

**Domain risk of bias: Low**

**Rationale:** The prospective cohort design and explicit inclusion/exclusion criteria are appropriate for model development. The main limitation is single-centre convenience sampling, which is more relevant to generalisability/applicability than internal selection bias.

**Applicability concern: Low**

**Applicability rationale:** The included population matches the study’s own target population (elective geriatric orthopedic surgery patients managed in PACU), although generalisability to emergency surgery and other centres is limited and is acknowledged in the manuscript.

## Domain 2. Predictors

**Description:** Final predictors: preoperative anxiety, cognitive function, dexmedetomidine use, intraoperative hypothermia, intraoperative blood loss, and immediate postoperative NRS pain score.

| **Question** | **Judgement** | **Manuscript basis** | **Rationale** |
| --- | --- | --- | --- |
| 2.1 Were predictors defined and assessed similarly for all participants? | Y | Methods 2.3–2.4, pp.3–4 | Predictor definitions and collection procedures were prespecified and applied to the cohort using questionnaires/scales, anesthesia information systems, and electronic records. |
| 2.2 Were predictor assessments made without knowledge of outcome data? | Y | Methods 2.4, p.4 | All final predictors were available before the first emergence-agitation assessment: preoperative/intraoperative variables preceded PACU outcome assessment, and NRS was obtained within 5 minutes whereas EA assessment started at 10 minutes. |
| 2.3 Are all predictors available at the time the model is intended to be used? | Y | Discussion 4.3 and Limitations, pp.10–11 | The model is explicitly positioned for use after the initial PACU assessment; all predictors are available by that time. |

**Domain risk of bias: Low**

**Rationale:** Predictors are clinically available and temporally aligned with the revised early-PACU use case. Dexmedetomidine is treated as an intraoperative exposure/predictive feature, not as evidence of a causal treatment effect.

**Applicability concern: Low**

**Applicability rationale:** Predictor definitions and timing match the stated early-PACU prediction question.

## Domain 3. Outcome

**Description:** Emergence agitation defined by SAS score ≥5, assessed first at 10 minutes after PACU admission and every 10 minutes thereafter throughout the PACU stay.

| **Question** | **Judgement** | **Manuscript basis** | **Rationale** |
| --- | --- | --- | --- |
| 3.1 Was the outcome determined appropriately? | Y | Methods 2.3.2 and 2.4, p.4 | A validated agitation scale (SAS) and a prespecified threshold were used. |
| 3.2 Was a prespecified or standard outcome definition used? | Y | Methods 2.3.2, p.4 | SAS ≥5 was specified as the criterion for emergence agitation. |
| 3.3 Were predictors excluded from the outcome definition? | Y | Methods 2.3–2.4, pp.3–4 | Predictors are not components of the SAS outcome definition. |
| 3.4 Was the outcome defined and determined similarly for all participants? | Y | Methods 2.4, p.4 | A uniform assessment schedule and threshold are reported for the cohort. |
| 3.5 Was the outcome determined without knowledge of predictor information? | NI | Methods 2.4, p.4 | The manuscript does not report blinding of outcome assessors to predictor information. |
| 3.6 Was the time interval between predictor assessment and outcome determination appropriate? | Y | Methods 2.4; Discussion 4.3, pp.4,10 | The last predictor (initial NRS) was obtained within 5 minutes; EA assessment began at 10 minutes and continued thereafter. |

**Domain risk of bias: Unclear**

**Rationale:** Outcome definition and timing are clear and standardized, but blinding of outcome assessors to predictor information is not reported. If assessors were blinded (or had no access to predictor information), this should be stated and this domain could potentially be judged low risk.

**Applicability concern: Low**

**Applicability rationale:** The outcome definition, timing, and PACU setting match the stated prediction question.

## Domain 4. Analysis

**Description:** Total N=612 with 169 EA events. The data were randomly split 70/30 (training n=430; test n=182). The training set had approximately 119 EA events and the test set approximately 50. Nineteen candidate predictors were considered; LASSO followed by multivariable logistic regression selected six predictors. Six algorithms were tuned using 5-fold cross-validation plus grid search; performance was evaluated using discrimination, calibration, and DCA. Missing data were addressed using mean/median imputation for <5% missingness and MICE for ≥5% missingness.

| **Question** | **Judgement** | **Manuscript basis** | **Rationale** |
| --- | --- | --- | --- |
| 4.1 Were there a reasonable number of participants with the outcome? | PN | Methods 2.2, 2.6–2.7; Results 3.1 | The development set contained ~119 events for 19 candidate predictors and several ML algorithms. Sample-size planning used EPV≥10 based on an anticipated final set of ≤10 predictors rather than the full candidate set and algorithmic complexity; therefore, overfitting risk cannot be excluded. |
| 4.2 Were continuous and categorical predictors handled appropriately? | Y | Methods 2.5–2.6, p.5 | Continuous variables were standardized and categorical variables encoded. |
| 4.3 Were all enrolled participants included in the analysis? | Y | Methods 2.5; Results 3.1 | All 612 included participants were analysed after prespecified handling of missing predictor data. |
| 4.4 Were participants with missing data handled appropriately? | PY | Methods 2.5, p.5; Supplementary Table S1 | MICE was used for variables with ≥5% missingness, while mean/median imputation was used for lower levels. This is broadly reasonable, although simple imputation can underestimate uncertainty. |
| 4.5 Was selection of predictors based on univariable analysis avoided? | Y | Methods 2.6, p.5 | Predictor selection used LASSO followed by multivariable logistic regression rather than univariable screening. |
| 4.6 Were complexities in the data accounted for appropriately? | Y / N/A | Study design | The outcome is a binary PACU event without censoring, competing risks, or control sampling requiring special handling. |
| 4.7 Were relevant model performance measures evaluated appropriately? | Y | Methods 2.7; Results 3.3 | Discrimination, calibration (including Brier score, intercept, slope), and clinical utility (DCA) were evaluated; AUC confidence intervals were reported. |
| 4.8 Were model overfitting and optimism in model performance accounted for? | PY | Methods 2.7, pp.5–6 | 5-fold cross-validation was used for hyperparameter tuning and an internal hold-out test set was used for performance evaluation. However, this remains internal validation and the test set was also used to compare/select the optimal algorithm. |
| 4.9 Do predictors and their assigned weights in the final model correspond to the multivariable analysis/model-fitting results? | PY | Methods 2.6–2.7 | The six selected predictors were fixed across models and each algorithm was fitted on the training set. For nonlinear learners, “weights” are not simple regression coefficients, but the fitted model reflects the training procedure. |

**Domain risk of bias: High**

**Rationale:** The principal concern is the number of outcome events relative to the 19 candidate predictors and the complexity of comparing/tuning six algorithms. In addition, the linear feature-selection pipeline may constrain nonlinear learners. Internal cross-validation and a hold-out set reduce, but do not eliminate, overfitting/optimism. Under PROBAST, any high-risk domain yields an overall high risk of bias.

# Step 4. Overall assessment

| **Model** | **Participants** | **Predictors** | **Outcome** | **Analysis** | **Overall risk of bias** |
| --- | --- | --- | --- | --- | --- |
| RF | Low | Low | Unclear | High | High |
| LR | Low | Low | Unclear | High | High |
| SVM | Low | Low | Unclear | High | High |
| KNN | Low | Low | Unclear | High | High |
| MLP | Low | Low | Unclear | High | High |
| XGBoost | Low | Low | Unclear | High | High |

**Overall applicability concern: Low**

**Summary:** Participants, predictor timing, and outcome definition are aligned with the stated early-PACU use case. The major limitation is methodological rather than applicability-related: this is a single-centre development study with only internal validation and a relatively modest number of outcome events for the candidate predictor set and multi-algorithm modelling workflow. PROBAST further advises caution for development studies without external validation unless based on a very large dataset with strong internal validation; therefore, the overall risk-of-bias judgement is High.
