# Supplementary material for: Development and validation of a machine learning-based prediction model for emergence agitation in geriatric patients undergoing orthopedic surgery: a prospective cohort study
Source: Front Med (Lausanne). 2026 Oct 14;13:1903005. doi: 10.3389/fmed.2026.1903005 (PMC13713869; doi:10.3389/fmed.2026.1903005)
Supplement: Supplementary file 3 [file Table3.docx]

**Supplementary Table S1.** Summary of missing data and imputation strategies for predictor variables in the training set.

| **Variables** | **Type** | **Missing (%)** | **Imputation Methods** |
| --- | --- | --- | --- |
| Intraoperative blood loss | Continuous | 6.28 | Multiple Imputation by Chained Equations |
| Preoperative albumin | Continuous | 1.40 | Median Imputation |
| Preoperative hemoglobin | Continuous | 0.70 | Median Imputation |
| Age | Continuous | 0.00 | None |
| BMI | Continuous | 0.00 | None |
| Preoperative anxiety | Continuous | 0.00 | None |
| Operative time | Continuous | 0.00 | None |
| NRS | Continuous | 0.00 | None |
| Gender | Categorical | 0.00 | None |
| Hypertension | Categorical | 0.00 | None |
| Diabetes | Categorical | 0.00 | None |
| Coronary Heart Disease | Categorical | 0.00 | None |
| Cognitive function | Categorical | 0.00 | None |
| ASA | Categorical | 0.00 | None |
| Dexmedetomidine use | Categorical | 0.00 | None |
| Type of surgery | Categorical | 0.00 | None |
| Intraoperative hypotension | Categorical | 0.00 | None |
| Intraoperative hypothermia | Categorical | 0.00 | None |
| Indwelling urinary catheter | Categorical | 0.00 | None |
