# Supplementary material for: Development and validation of a machine learning-based prediction model for emergence agitation in geriatric patients undergoing orthopedic surgery: a prospective cohort study
Source: Front Med (Lausanne). 2026 Oct 14;13:1903005. doi: 10.3389/fmed.2026.1903005 (PMC13713869; doi:10.3389/fmed.2026.1903005)
Supplement: Supplementary file 4 [file Table4.docx]

**Supplementary Table S2.** Hyperparameter search spaces and final selected parameters for the six machine learning models.

| Model | Hyperparameter | Search Space | Optimal Value |
| --- | --- | --- | --- |
| RF | Mtry | (2, 3, 4, 5) | 2 |
|  | Min.node.size | (5, 10, 15, 20, 25, 30) | 15 |
|  | Num.trees | (100, 200, 300, 400, 500) | 400 |
|  | Max.depth | (3, 5, 8) | 5 |
| LR | Alpha | (0, 0.1, 0.5, 1) | 0 |
|  | Lambda | exp(seq(-5, 2, length = 100)) | 0.2154 |
| SVM | Sigma | 2^seq(-10, 3, length = 10) | 0.0197 |
|  | C | 2^seq(-5, 5, length = 10) | 14.814 |
| KNN | K | seq(3, 30, by = 2) | 13 |
| MLP | Epochs | (50, 100, 150, 200, 250) | 50 |
|  | Hidden Layer Size | (5, 10, 15, 20) | 10 |
|  | Learning Rate Decay | (0.0001, 0.001, 0.01, 0.1) | 0.1 |
| XGBoost | Nrounds | (50, 100, 150) | 150 |
|  | Max.depth | (3, 5, 7) | 3 |
|  | Learning Rate | (0.01, 0.05, 0.1) | 0.05 |
|  | Gamma | (0, 1, 5) | 1 |
|  | Colsample.bytree | (0.6, 0.8, 1.0) | 0. 6 |
|  | Min.child.weight | (1, 3, 5) | 3 |
|  | Subsample | (0.6, 0.8, 1.0) | 0.8 |
