# Supplementary material for: Development and validation of a machine learning-based prediction model for emergence agitation in geriatric patients undergoing orthopedic surgery: a prospective cohort study
Source: Front Med (Lausanne). 2026 Oct 14;13:1903005. doi: 10.3389/fmed.2026.1903005 (PMC13713869; doi:10.3389/fmed.2026.1903005)
Supplement: Supplementary file 5 [file Table5.docx]

**Supplementary Table S3.** Calibration intercept and slope of the six models in the training set and test set.

| **Model** | **Training set** | | **Test set.** | |
| --- | --- | --- | --- | --- |
|  | Intercept | Slope | Intercept | Slope |
| RF | -0.066 | 1.240 | -0.024 | 1.100 |
| LR | -0.198 | 1.720 | -0.130 | 1.450 |
| SVM | -0.012 | 1.070 | 0.044 | 0.869 |
| KNN | -0.022 | 1.160 | 0.043 | 0.901 |
| MLP | -0.015 | 1.040 | 0.073 | 0.762 |
| XGBoost | -0.066 | 0.947 | -0.020 | 0.835 |
